# Uncovering an Easily Overlooked Cause of Dyspnea: Partial Anomalous Pulmonary Venous Connection of the Right Pulmonary Vein to the Superior Vena Cava Leading to Right Heart Enlargement

**DOI:** 10.7759/cureus.35369

**Published:** 2023-02-23

**Authors:** Victor H Molina-Lopez, Christina Arraut-Hernandez, Carlos Nieves-La Cruz, Alvin A Almodovar-Adorno, Jaime Rivera-Babilonia

**Affiliations:** 1 Cardiology, Veteran Affairs Caribbean Healthcare System, San Juan, PRI; 2 Interventional Cardiology, Veteran Affairs Caribbean Healthcare System, San Juan, PRI; 3 Interventional Radiology, Veteran Affairs Caribbean Healthcare System, San Juan, PRI

**Keywords:** adult congenital heart disease (achd), cardiac shunt, anomalous pulmonary venous drainage, partial anomalous pulmonary circulation, congenital cardiac anomaly

## Abstract

This case report describes a rare variant of partial anomalous pulmonary venous connections (PAPVCs) in a patient who presented with an insidious progression of dyspnea on exertion as an adult, leading to the diagnosis of PAPVC. The patient had an anomalous right upper pulmonary vein connecting to an anomalous pulmonary-azygos trunk that connected to the cranial superior vena cava (SVC), producing a large left-to-right extracardiac shunt. The diagnosis of PAPVC was made after evaluating for causes of right heart chamber enlargement. This case highlights the importance of considering PAPVC as a potential cause of unclear etiology for exertional dyspnea, right-sided chamber enlargements, and intact atrial septum. The onset and severity of symptoms in patients with PAPVC depend on various factors, including the number of pulmonary veins, site of connection, pulmonary vascular resistance, atrial compliance, and the presence of other congenital heart defects. Therefore, clinicians should maintain a high level of suspicion for PAPVC in patients with these types of symptoms.

## Introduction

Partial anomalous pulmonary venous connections (PAPVCs) are rare congenital defects in which one or more of the pulmonary veins connect to a systemic vein rather than to the left atrium, producing a left-to-right extracardiac shunt. This anomaly has many variations; some can be clinically silent and found incidentally in imaging studies. Others may become symptomatic at younger ages. Some patients can develop right-sided volume overload if left untreated, eventually leading to cardiac remodeling [1-3]. Aside from a strong clinical suspicion, patient evaluation and diagnostic workup involve imaging studies to determine anatomical variants and right heart catheterization to estimate shunt fraction and right heart hemodynamics. Some patients may qualify for surgical correction of PAPVC to improve quality of life and morbidity. In this case, we present the case of a patient undergoing evaluation for the complaint of dyspnea, which was found incidentally with imaging studies with an unclear etiology for right-sided chamber enlargements and intact atrial septum, later confirmed to be a PAPVC. We will discuss the complex approach to the evaluation and management of patients with PAPVC.

## Case presentation

**Figure 1:**
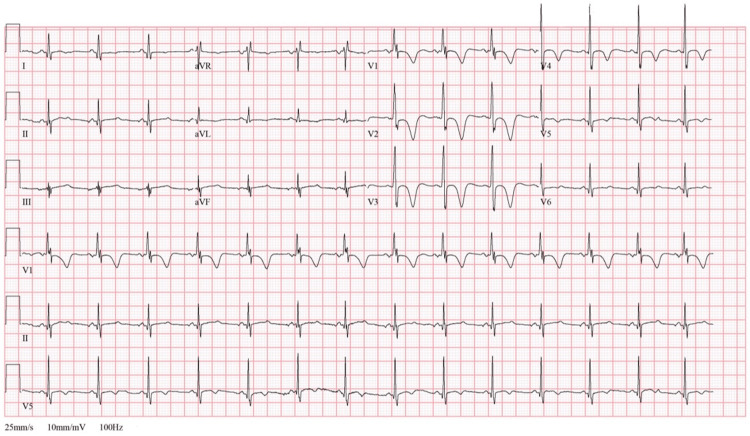
The 12-lead electrocardiogram demonstrating the right ventricular strain pattern. The electrocardiogram revealed normal sinus rhythm, incomplete right bundle branch block pattern with dominant R wave in V1 > 7 mm tall, S1S2S3 in inferior leads, and ST depressions with marked T-wave inversions in V1-V4. Notching of the QRS complexes can be appreciated in leads II, III, and aVF.

A 55-year-old man presented to the outpatient clinic complaining of worsening dyspnea on exertion with ordinary physical activity. The patient was a non-diabetic male, non-smoker, with hyperlipidemia, hypertension, and chronic kidney disease 3a attributed to hypertensive nephropathy. His home medications included losartan 100 mg daily, hydrochlorothiazide 12.5 mg daily, amlodipine 10 mg daily, and atorvastatin 20 mg daily. His family history was unremarkable for congenital disorders. On physical examination, the chest appeared normal without lifts, heaves, or thrills. The heart rate and rhythm were normal. No murmurs, gallops, or rubs were auscultated. S1 and S2 were normal, and no S3 or S4 were present. However, there was mild jugular venous distension and hepatojugular reflux. Extremities showed trace pitting edema. An initial 12-lead electrocardiogram (ECG) showed normal sinus rhythm and a right ventricular strain pattern, as shown in Figure 1.

**Figure 2:**
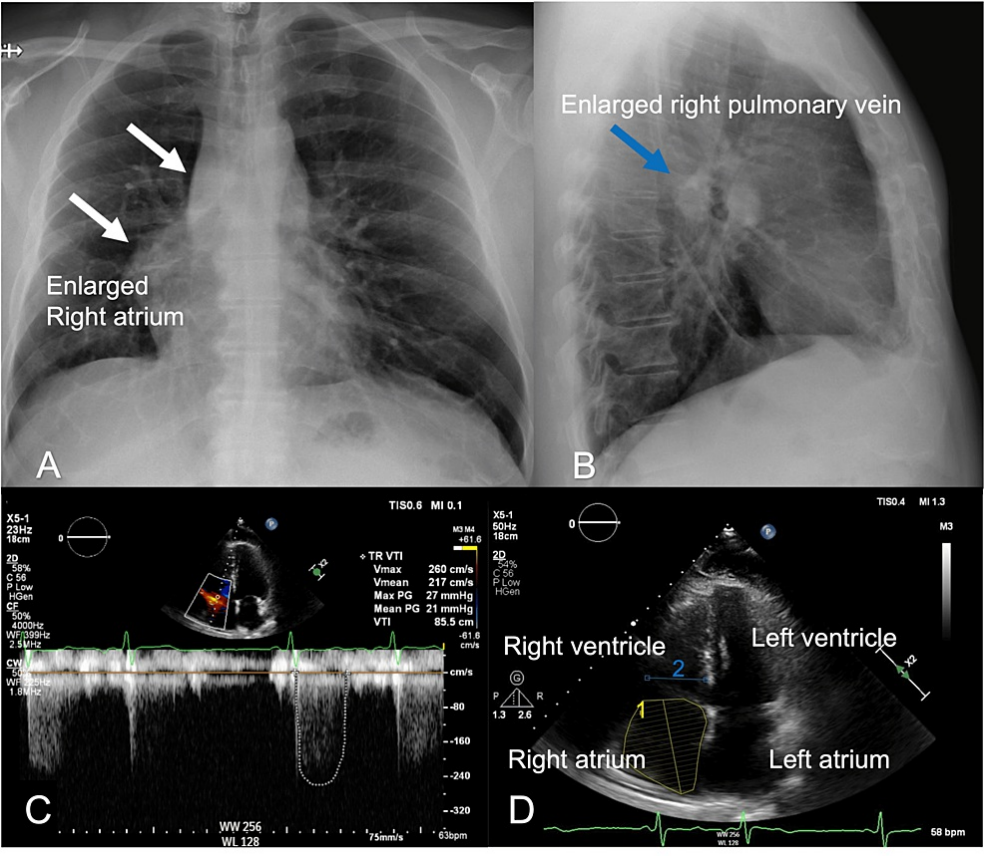
(A) Posteroanterior and (B) lateral chest radiograph demonstrating an enlarged right heart border (white arrows) and enlarged right pulmonary vein (blue arrow). Transthoracic echocardiogram with four-chamber apical views revealing (C) mild tricuspid regurgitation and (D) enlargement of right heart chambers.

Laboratory results revealed hemoglobin of 16 g/dL. N-terminal pro-B-type natriuretic peptide (NT-proBNP) was elevated but stable at a 1,180 pg/mL baseline. High-sensitive cardiac troponin T (hs-cTnT) was mildly elevated but stable at his baseline. Arterial blood gases revealed no hypoxemia at room air. Lateral and posteroanterior (PA) chest radiography was remarkable for an enlarged right heart border, including the right atrium and the superior vena cava (SVC) border, widening of the right paratracheal stripe, with increased and centralized pulmonary vascularity (Figures 2A, 2B). The two-dimensional transthoracic echocardiogram (2D-TTE) demonstrated a normal left ventricular function with end-diastolic septal flattening and mild right ventricle dilatation, borderline right atrial enlargement, and mild tricuspid regurgitation (Figures 2C, 2D). Using 2D-TTE, the right atrial pressure (RAP) was estimated at 10 mmHg, the mean pulmonary artery pressure (mPAP) was 31 mmHg, and the right ventricular systolic pressure (RVSP) was estimated at 37 mmHg. Agitated saline combined with physiologic maneuvers to provoke right-to-left shunting did not reveal a patent foramen ovale (PFO) or an atrial septal defect (ASD).

**Figure 3:**
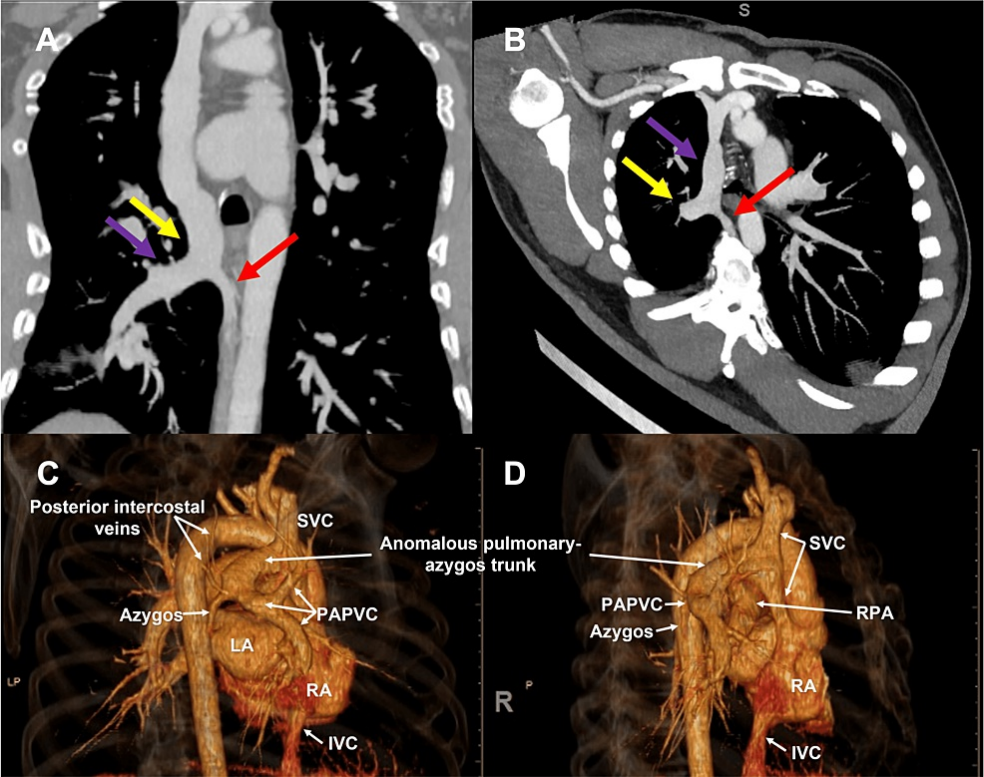
Contrast-enhanced chest CT revealing the PAPVC with oblique views (A and B) describing the junction between the anomalous right pulmonary vein (purple arrow) to the cranial aspect of the SVC (yellow arrow), as well as the enlarged azygous vein (red arrow) connecting to the anomalous pulmonary vein. Usually, the right superior pulmonary vein drains the right upper and middle lobes. The right inferior pulmonary vein drains the right lower lobe. However, this patient lacked any functional venous connection to the left atrium from the right lung. Three-dimensional CT reconstruction (C and D) demonstrates the right PAPVC to the SVC with the azygous vein and intercostal veins draining into the right anomalous pulmonary vein. RA = right atrium; IVC = inferior vena cava; SVC = superior vena cava; PAPVC = partial anomalous pulmonary venous connection

A chest CT with intravenous (IV) contrast (Figures 3A, 3B) and three-dimensional reconstruction (Figures 3C, 3D) revealed enlargement of the right atrium and right ventricle with a partial anomalous venous connection of a large dominant right upper pulmonary vein draining the right-sided lung segments, connecting to the cranial aspect of the SVC. The left lower pulmonary vein was atretic with hypoplasia of the lower lung. He also had an anomalous connection of the azygos vein into the PAPVC, which led to a mixing of deoxygenated systemic blood to the oxygenated pulmonary venous flow through the azygous venous drainage system. The left atrium received circulation from the left lung through the left superior and left inferior pulmonary veins.

**Figure 4:**
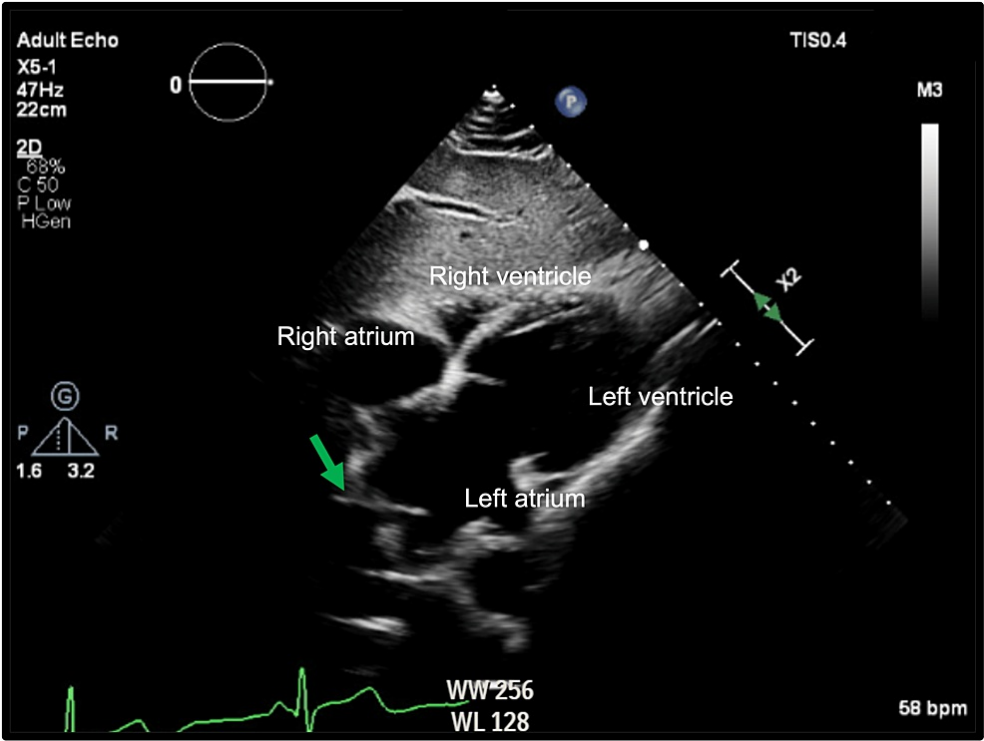
Transthoracic echocardiogram subxiphoid view demonstrating the baffle repair conduit joining the left atrium (green arrow).

The pulmonary function test (PFT) was normal. Continuous 24-hour external cardiac telemetry monitor showed atrial arrhythmia with 4,328 premature atrial contractions (PACs) in 24 hours, consistent with a 6.6% PAC burden. Coronary angiography demonstrated non-obstructive coronary artery disease, left ventricular end-diastolic pressure of 18 mmHg, and left ventricular ejection fraction of 65% with no wall motion abnormalities. Right heart hemodynamics revealed a RAP of 11 mmHg, pulmonary artery wedge pressure of 16 mmHg, and mPAP of 21 mmHg. Systolic pulmonary artery pressure was <50% of systemic pressure, and pulmonary vascular resistance was <33% of systemic vascular resistance. The oxygen saturation runs demonstrated a significant step-up from the right innominate vein of 68.5% to the high SVC of 98.6% with a pulmonary-to-systemic flow shunt ratio (Qp:Qs) of 2.3 and 58% left-to-right shunt. TEE or gated cardiac magnetic resonance imaging failed to show a sinus venosus ASD or other intracardiac shunts. The patient was then referred to a cardiothoracic surgeon for surgical repair. He underwent surgical repair via midline sternotomy, reconfiguring the anomalous pulmonary vein circulation to the left atrium with an intracardiac baffle through a surgically created ASD. Follow-up 2D-TTE (Figure 4) performed six months postoperatively revealed normalized right atrial and ventricular volumes. Postoperative ECG had resolution of ST depressions and T-wave inversions in V1-V4 and S1S2S3 pattern but persisted with incomplete right bundle branch block pattern and fragmented QRS complex on inferior leads.

## Discussion

Our case describes a PAPVC variant that comprises an anomalous right upper pulmonary vein that connects to the cranial aspect of the SVC, producing a left-to-right shunt with a large pulmonary-to-systemic shunt (Qp:Qs). The left lower pulmonary vein was atretic with hypoplasia of those lung segments. He also had an anomalous connection of the azygos vein into the PAPVC, which led to a mixing of deoxygenated systemic blood to the oxygenated pulmonary venous flow through the azygous venous drainage system. This patient remained without significant symptoms most of his young adult life with good exercise tolerance, presenting with an insidious progression of dyspnea on exertion as an adult. In this case, the PAPVC was found after evaluating for causes of right heart chamber enlargement. In patients with PAPVC and partial anomalous pulmonary venous return (PAPVR), the onset and severity of symptoms depend on many factors, including the number of pulmonary veins, site of connection, pulmonary vascular resistance, atrial compliance, the presence of ASDs, or the co-existence with other congenital heart defects. Clinicians should entertain a high level of suspicion for the presence of PAPVCs in patients with unclear etiology for exertional dyspnea, right-sided chamber enlargements, and intact atrial septum [1-4].

PAPVCs occur due to the anomalous connection of a pulmonary vein into a systemic vein, mixing oxygenated blood from the pulmonary circulation with the systemic venous blood before returning to the right side of the heart. Similarly, PAPVR refers to mixing oxygenated blood from the pulmonary venous circulation into the right atrium. PAPVC infrequently occurs, with a prevalence of around 0.2-0.7% in adults and exhibiting a broad spectrum of anomalous variations [1-3]. In a series by Alsoufi et al., the most common types of PAPVC are right-sided (90%), left-sided (7%), and bilateral (2%). Anomalous veins were partial in 79% and involved the entire lung in 21% of patients. The most common type was right PAPVC into the SVC in 74%, with 87% associated with sinus-venous ASD, followed by right PAPVC into the right atrium in 12%, left PAPVC into the innominate vein in 9%, and scimitar syndrome in 6% [3].

The PAPVC variant demonstrated in this case can be possible due to similar embryologic origins between the azygos venous and pulmonary venous systems. PAPVCs occur from the failure of the primitive lung venous system to regress properly. During early embryologic development, the primordial lung buds drain through a vascular bed to the cardinal veins, developing into systemic veins. Usually, the right anterior cardinal and common cardinal veins form the SVC, and the left anterior cardinal vein regresses [4,5]. The azygos vein arises from the right supracardinal vein, while the left supracardinal vein becomes the hemiazygos vein [6].

PAPVCs can be clinically silent in adults and sometimes found incidentally after imaging studies [1,7,8]. This abnormal systemic venous connection act as an ex-extracardiac left-to-right shunt due to the recirculation of oxygenated blood back to the right heart. Over time, this increase in pulmonary blood flow can lead to remodeling the central venous circulation, pulmonary venous circulation, and right heart structures. If severe, pulmonary arterial hypertension and right ventricular failure can occur [7,8].

When evaluating patients for PAPVC, chest radiography has limited sensitivity but can show abnormal right heart contour and enlargement of the paratracheal stripe. Chest CT imaging with IV contrast can efficiently delineate central vessels and pulmonary vasculature anatomic variations. Gated cardiac CT or MRI angiography with three-dimensional reconstruction may help quantify chamber volumes and valve regurgitation, estimate shunt ratio, and delineate anomalous venous connection origin, course, and anastomosis. ECG findings can be non-specific but may suggest right heart strain or remodeling. For patients with complaints of dizziness or a history of syncope, Holter monitoring can be a practical tool to assess for conduction disorders and atrial arrhythmias [9,10]. TTE can help estimate right heart pressures and provide structural information such as right ventricular and atrial volumes, evaluate for ASDs (typically sinus venosus or secundum defects), and provide a non-invasive assessment of the shunt. TEE can be further used to evaluate for PFO or ASDs. Right and left heart catheterization can provide the most accurate method for assessing pulmonary and systemic flow shut ratios [8]. Physiologically, a PAPVC behaves similarly to an ASD; however, unlike ASDs, anomalous pulmonary veins represent obligate left-to-right shunts. They may occur in isolation or with a concomitant ASD. Anomalous pulmonary vein cannulation is crucial in evaluating pulmonary vein oxygen saturation and Qp.

**Figure 5:**
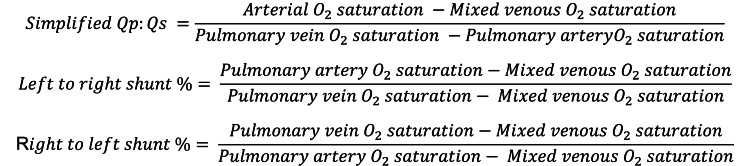
Equations used for the calculation of pulmonary-to-systemic flow ratio (Qp:Qs), as well as the left-to-right shunt percentage and right-to-left shunt percentage. Qp = pulmonary flow; Qs = systemic flow; O_2_ = oxygen

Determining the anatomy by imaging may help guide clinicians to the most appropriate site for sampling mixed venous oxygen saturation [11]. In our patient, the most representative measurement of mixed venous blood was obtained from the left innominate vein, which was secondary to the re-distribution of oxygenated blood from the right pulmonary circulation through the azygous system and inferior vena cava. In cases of ≥2 right-sided anomalous veins or a large left upper and lingular vein may result in Qp/Qs between 1.5 and 2.0, whereas a single right-sided anomalous vein or isolated left upper pulmonary vein tends to be associated with Qp:Qs of ≤1.5 [10]. Figure 5 demonstrates the equations commonly used for calculating shunt fraction and step-up values expected in typical cases of PAPVCs [12].

## Conclusions

Anomalous pulmonary vein connections are rare congenital heart defects in which one or more pulmonary veins do not connect normally to the left atrium. Instead, they may connect with the right atrium, other cardiac veins, or the superior or inferior vena cava. APVCs can cause complications such as arrhythmia, pulmonary hypertension, and even heart failure. Treatment often involves open-heart surgery to repair the abnormal connection and restore normal blood flow throughout the heart. Strong clinical suspicion is needed for an adequate diagnosis in patients with congenital anomalies, which may not present with symptoms at an early age. This study helps to highlight the importance of early diagnosis of partial anomalous pulmonary vein connections as early corrective surgery can reduce the risk of long-term complications such as heart failure.
